# Intracoronary Pacing during “Chimney Technique” in Transcatheter Aortic Valve-in-Valve Implantation: An Alternative Temporary Rapid Ventricular Stimulation?

**DOI:** 10.3390/jcdd10080341

**Published:** 2023-08-08

**Authors:** Alessandro Cafaro, Francesco Rizzo, Dionigi Fischetti, Luca Quarta, Marco Mussardo, Alessandro Mandurino-Mirizzi, Antonio Tondo, Marco Matteo Ciccone, Fortunato Iacovelli, Giuseppe Colonna

**Affiliations:** 1 Division of Cardiology, “V. Fazzi” Hospital, 73100 Lecce, Italy; dr.alessandrocafaro@libero.it (A.C.); dionigi.fischetti@gmail.com (D.F.); lucaquarta2@gmail.com (L.Q.); m.mussardo@gmail.com (M.M.); ale.mandurinomirizzi@gmail.com (A.M.-M.); antonio.tondo66@virgilio.it (A.T.); giuseppe.colonna@tin.it (G.C.); 2 Division of University Cardiology, Cardiothoracic Department, Policlinico University Hospital, 70124 Bari, Italy; marcomatteo.ciccone@uniba.it (M.M.C.); fortunato.iacovelli@gmail.com (F.I.); 3 Division of Cardiology, “SS. Annunziata” Hospital, 74121 Taranto, Italy

**Keywords:** transcatheter aortic valve implantation, valve-in-valve, chimney technique, coronary artery occlusion, intracoronary pacing

## Abstract

Temporary rapid ventricular pacing (TRVP) is required during transcatheter aortic valve implantation (TAVI) in order to reduce cardiac output and to facilitate balloon aortic valvuloplasty, prosthesis deployment, and post-deployment balloon dilation. The two most frequently used TRVP techniques are right endocardial (RE)-TRVP and retrograde left endocardial temporary rapid ventricular pacing (RLE)-TRVP. The first one could be responsible for cardiac tamponade, one of the most serious procedural complications during TAVI, while the second one could often be unsuccessful. Intracoronary (IC)-TRVP through a coronary guidewire has been described as a safe and efficient procedure that could avoid such complications. We describe two clinical cases in which IC-TRVP has been effectively used during valve-in-valve TAVI with coronary protection via the “chimney technique”, after unsuccessful RLE-TRVP.

## 1. Case 1

Case 1 is about a 68-year-old female patient scheduled for valve-in-valve transcatheter aortic valve implantation (ViV-TAVI) after heart team discussion. She complained of New York Heart Association (NYHA) functional class III in severe aortic prosthetic dysfunction (aortic valve area = 0.8 cm^2^). A stentless porcine bioprosthesis Freestyle™ (Medtronic, Minneapolis, MN, USA) 21 mm was implanted 12 years before because of severe aortic regurgitation. Cardiovascular risk factors were hypertension, hypercholesterolemia, and former smoking. She also had a history of peripheral arterial disease, previous transient cerebral ischemia and coronary artery disease already treated with coronary artery by-pass grafting, i.e., left internal mammary artery (LIMA) on left anterior descending (LAD) coronary artery and saphenous vein graft on posterior descending artery. A percutaneous coronary intervention (PCI) with rotational atherectomy on left main coronary artery (LMCA) and LAD was recently performed, because of LIMA graft chronic total occlusion. After 1.5 and 1.75 mm burr passages at 160,000 rounds per minute, the mid- and proximal LAD and LMCA heavy calcified stenosis were treated with three sirolimus-eluting stents (3.0 × 18, 3.5 × 33, and 4.0 × 24 mm, respectively) implantation. Intracoronary imaging-guided postdilation until 5.0 mm of the LMCA was performed, with 1 mm strut protrusion into the ascending aorta. A permanent dual chamber pacemaker was implanted one month before TAVI because of sinoatrial block. Other comorbidities were chronic hypochromic microcytic anemia, paroxysmal atrial fibrillation, moderate chronic kidney disease, and anxious-depressive syndrome. Left ventricular ejection fraction (LVEF) was normal. The European System for Cardiac Operative Risk Evaluation (EuroSCORE) II and Society of Thoracic Surgeons predictive risk of mortality (STS-PROM) score were 8.5% and 4%, respectively. Because of heavy calcifications and tortuosity of right ilio-femoral arterial axis, the left side was chosen as main transfemoral access route. A polytetrafluoroethylene-coated, stainless steel 0.035″ wire was inserted into the left ventricle through an Amplatz left (AL) 1 diagnostic catheter. The risk of LMCA occlusion was considered high, due to the valve type, small area of the sinuses of Valsalva, and low valve-to-LMCA height (about 5 mm).

Prophylactic “chimney snorkel” strategy was performed. A 7 French (F) Judkins left 4-curved coronary guiding catheter was advanced till LMCA ostium. A hydrophilic coating over spring coil 0.014″ guidewire was advanced till distal LAD and loaded with a zotarolimus-eluting stent 4.0 × 38 mm that was advanced with mild friction due to previous PCI. The stent length was chosen in order to avoid any further crossing difficulty, to increase radial force with struts overlap, and to ensure a 10–12 mm protrusion enough to overtake both the sinotubular aortic junction and the frame of the intended transcatheter heart valve to implant.

**Figure 1:**
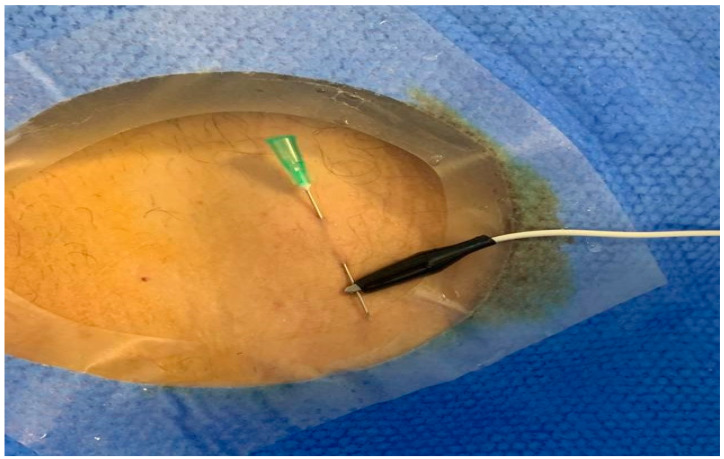
Adaptive alligator clip connecting the guidewire to the pulse generator used as unipolar cathode and the skin needle used as indifferent electrode.

An attempt of retrograde left endocardial temporary rapid ventricular pacing (RLE-TRVP) was unsuccessful. In order to avoid additional vein access and to reduce the potential risk for right ventricular perforation due to right endocardial (RE)-TRVP, an intracoronary (IC)-TRVP was effectively performed (Figure 1): an adaptive alligator clip connecting the guidewire to the pulse generator was used as unipolar cathode, and a skin needle was used as indifferent electrode.

**Figure 2:**
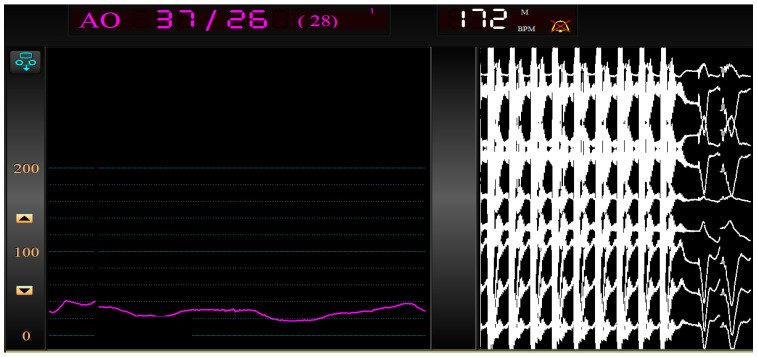
Left pressure wave flattening, via effective IC-TRVP.

A 20 V maximum unipolar output current erogation was used in order to obtain a pacing of 180 beats per minute (Figure 2). No significant issues on coronary vessels, such as flow retrieve or spasm, occurred.

After the pacing test, a balloon-expandable Edwards Sapien 3™ (Edwards Lifesciences Inc., Irvine, CA, USA) 23 mm valve was directly implanted with no residual significant paravalvular leak and an immediate mean transvalvular pressure gradient of 6 mmHg. Valve positioning was not hampered by LMCA previously implanted stent.

**Figure 3:**
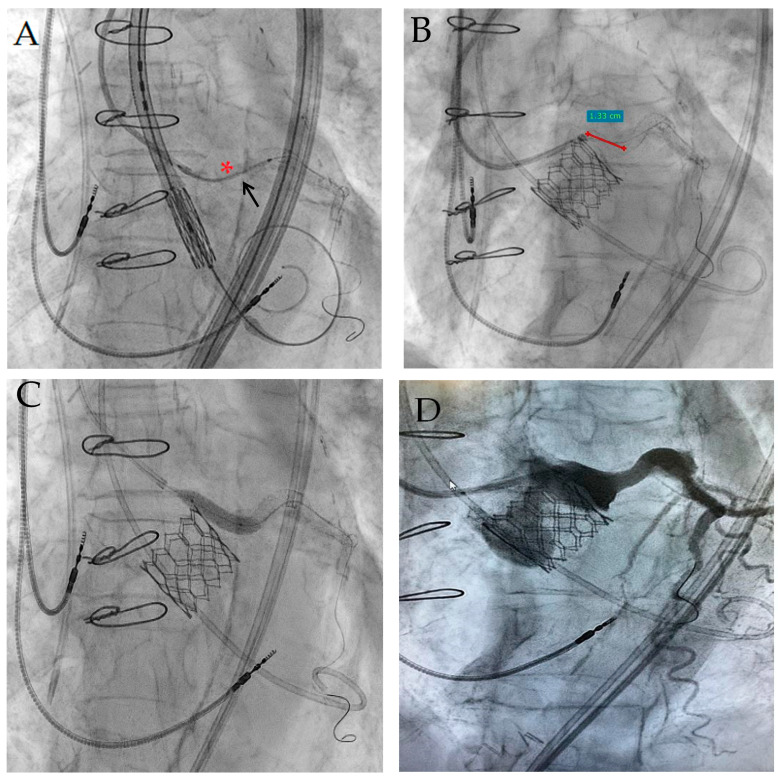
“Chimney stent” (*asterisk*) positioning in LMCA and previously implanted LMCA edge aortic protrusion (*arrow*) immediately before valve implantation (**A**); “Chimney stent” protrusion into aorta (**B**) and postdilation (**C**); final left coronary angiography after Sapien 3 valve implantation (**D**).

During valve inflation, the coronary guiding catheter was temporarily retrieved, while the new stent was kept in position with sufficient aortic protrusion, ready for immediate implantation (Figure 3A). After valve delivery, a subselective angiography showed reduced LMCA flow due to a displaced surgical bioprosthesis leaflet. The stent was then pulled back, expanded, and postdilated until 20 atmospheres through a 4.5 mm non-compliant balloon (Figure 3B). The “chimney stent” protrusion into the ascending aorta was about 13 mm (Figure 3C), which was close to the estimated implanted valve height of 14 mm. A contemporary “kissing” valve and stent postdilation was not necessary because of an optimal result. Guidewire “stent recrossing test” confirmed the optimal stent placement, just as a chimney parallel to the last implanted valve (Figure 3D), preserving a possible LMCA access for further coronary interventions.

Four days after such a procedure, the patient had been successfully discharged on an oral anticoagulation therapy with apixaban and single antiplatelet therapy with clopidogrel.

## 2. Case 2

We report the case of an 84-year-old male patient scheduled for ViV-TAVI. Similarly to the former case, he was in NYHA functional class III because of severe aortic prosthetic dysfunction (peak/medium pressure gradients 88/60 mmHg). Cardiovascular risk factors were hypertension, dyslipidemia, overweight, and former smoking; he also referred family history of aortic aneurysm. In 2012, he underwent TAVI with a Direct Flow Medical^®^ (Direct Flow Medical Inc., Santa Rosa, CA, USA) 23 mm bioprosthesis, and soon after, a permanent pacemaker was implanted too. Three years later, the prosthetic valve was affected by thrombosis, successfully treated with unfractionated heparin administration. In August 2020, he underwent a PCI with drug-eluting stent implantation on LAD because of a chronic coronary syndrome. Other comorbidities were anemia and paroxysmal atrial fibrillation; LVEF was normal. EuroSCORE II and STS-PROM score were 5.5% and 3.5%, respectively.

Right arterial access was chosen as main transfemoral route. A pre-shaped stainless steel 0.035″ TAVI-dedicated wire was inserted into the left ventricle through an AL1 diagnostic catheter. A prophylactic “chimney snorkel” strategy was performed. The LMCA ostium was cannulated with a 7 F 4-curved left coronary guiding catheter. As in Case 1, a hydrophilic coating over spring coil 0.014” protection guidewire was inserted into the distal LAD and loaded with a zotarolimus-eluting stent 4.0 × 30 mm. Left femoral arterial access was chosen for insertion of an embolic protection device on the aortic arch.

**Figure 4:**
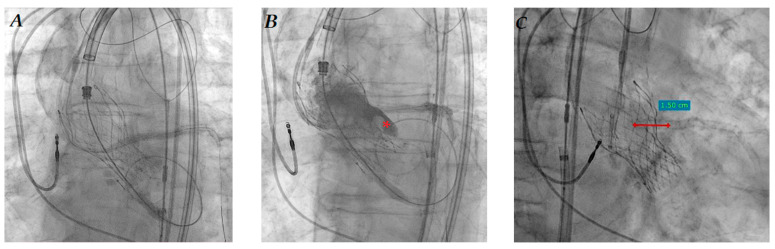
LMCA stent implantation (**A**); subselective coronary angiography after stent implantation with Direct Flow leaflet (asterisk) near LMCA ostium after Evolut R release (**B**); stent protrusion into the aorta (**C**).

RLE-TRVP through the 0.035″ wire resulted unsuccessful because of unstable and inconstant stimulation. IC-TRVP was then successfully obtained in the same way as that described in Case 1; there were no significant issues on coronary vessels. After the pacing test, a self-expanding Evolut R™ (Medtronic, Minneapolis, MN, USA) 23 mm bioprosthesis was directly implanted with no significant paravalvular leak and a mean transvalvular gradient of 20 mmHg. After valve deployment, the “chimney stent” was promptly implanted (Figure 4A) and properly postdilated with a 4.5 mm non-compliant balloon, because of significant LMCA flow obstruction due to Direct Flow leaflet dislocation (asterisk in Figure 4B): the stent protrusion into the aorta was about 15 mm (Figure 4C). The “stent recrossing test” with 0.014″ guidewire was successful.

The patient was successfully discharged one week after, with an appropriate apixaban and clopidogrel prescription.

## 3. Discussion

Along TAVI procedures, TRVP is required in order to reduce cardiac output, facilitating balloon aortic valvuloplasty (BAV), prosthesis deployment, and post-deployment balloon dilation. TRVP failure is known to be more often accompanied by valve malpositioning, and accounts for 11% of valve embolization cases [1]. Furthermore, cardiac tamponade is one of the most serious procedural complications during TAVI, with a reported incidence of 0.2–4.3% [2] and an associated mortality rate of 23.5% [3]. Reports suggest that in more than half of cases (52.9%), cardiac tamponade results from perforation of the right ventricle [2,3,4]. As a safer alternative to RE-TRVP, RLE-TRVP through a supportive 0.035″ wire has already been described along BAV and TAVI [5]. Nevertheless, Faurie et al. reported a rate of unsuccessful RE- and RLE-TRVP of 12.9% and 15.1%, respectively [6]. In our cases, RLE-TRVP was ineffective with all possible electrode settings, maybe because of the wires’ structure. Maneuvers on TAVI delivery system could also interfere with electrode placement on the wire tail.

IC-TRVP, with or without over-the-wire balloon coverage, has been previously described in animal models too [7,8]. In 2022, Heinroth et al. published their successful IC-TRVP experience with a double-wire technique in pig models, using a polytetrafluoroethylene-covered guidewire in the proximal segment of the vessel serving as indifferent anode, and another standard guidewire, advanced into the periphery of the same coronary artery and covered with a coronary balloon, as cathode [9].

Different types of modern coronary guidewires have been successfully tested in order to obtain IC-RTVP without pacing-related complications. The registered pacing resistance was low, and it varied from 12 to 31 ohms depending upon the wire distance from the tip [10].

In humans, this stimulation modality was initially used to detect myocardial viability, and it was first described in 1984 [11]. In six patients, O’Neill demonstrated that the IC-TRVP impedance and pacing thresholds were linked to viable myocardial segments in cardiac magnetic resonance performed before PCI. Pacing was achieved in 40 different coronary tracts; each site of IC stimulation was then compared to the corresponding tributary areas at cardiac magnetic resonance and to the presence, in the same segments, of an epicardial coronary stenosis ≥50%: the evidence of a myocardial scar without any stenosis reducing the coronary lumen more than 50% had significantly different conductance parameters during IC-TRVP [12]. In 2006, Heinroth et al. reported a successful LAD IC-TRVP in 90% of a total of 30 patients undergoing PCI, with low risk of transient coronary spasm [13]. Recently, Mallek et al. reported a case of PCI of a right coronary artery ostial lesion in which IC-TRVP allowed precise stent positioning and implantation [14]. Moreover, the efficacy and safety of the adjunctive IC pacing were tested during rotational atherectomy too [15,16].

Despite its promising data in experimental and clinical settings, this method has not gained general acceptance yet. In fact, to our knowledge, these are the first cases of successful IC-TRVP in TAVI setting. Another peculiar aspect is that our Case 2 is the second reported one of degenerated Direct Flow Medical bioprosthesis treated with a self-expanding transcatheter heart valve implantation, and it is absolutely the first with an Evolut R. In the only other case reported in the literature, a degenerated Direct Flow Medical bioprosthesis was fixed through a ViV implantation of an ALLEGRA™ bioprosthesis (Biosensors International LTD, Wilmington, DE, USA) [17].

Besides being an easy, safe, and stable alternative to RE- and RLE-TRVP, such an IC strategy could avoid the risk of both right ventricular perforation and infectious or hemorrhagic complications at the transfemoral venous access, especially in high-bleeding risk subjects, who represent the majority of TAVI patients.

Finally, IC-TRVP could be furtherly suggested in the case of coronary protection because of the high risk of acute coronary occlusion (CO). Indeed, the latter represents an uncommon but severe complication that occurs during or following <1% of TAVI procedures but carries a 30-day mortality risk of 40-to-50% in published series and registries [18,19]. Among the several risk factors for CO during TAVI, ViV implantation to treat degenerated stentless or stented —but with externally mounted leaflet— bioprostheses, could be considered the most predictive one [18,20,21]. The prophylactic “chimney snorkel” technique has been proven to prevent CO in selected settings [22]. An adequate coronary flow after valve deployment is not a sufficient parameter to decide about stent implantation during coronary protection. Indeed, delayed CO after ViV-TAVI has been reported in cases with high-risk features. In a recently reported series, 27.7% of patients presenting with delayed CO were only protected without stent release because of normal angiographic flow in LMCA after valve expansion, whereas 18.4% received an ostial (non-chimney/non-snorkel) stent [20]. So, during ViV-TAVI at high risk for CO, a low threshold for deploying the coronary stent is recommended. On the other hand, a substantial portion of the “chimney stent” needs to be hung into the ascending aorta, and ideally, the protrusion has to be enough to come above the highest portion of the sealed segment of the transcatheter bioprosthesis.

## 4. Conclusions

These are, to our knowledge, the first cases of successful IC-TRVP during TAVI. Further studies are needed to confirm the safety and feasibility of this preliminary evidence and maybe to extend IC-TRVP indication to all TAVI patients when coronary protection or PCI are needed, and alternative TRVP methods fail or result in greater risks.

## Data Availability

Data are accessible with the article (raw data are available upon individual request).
